# QSPR study on the octanol/air partition coefficient of polybrominated diphenyl ethers by using molecular distance-edge vector index

**DOI:** 10.1186/1752-153X-8-36

**Published:** 2014-06-10

**Authors:** Long Jiao, Mingming Gao, Xiaofei Wang, Hua Li

**Affiliations:** 1 College of Chemistry and Chemical Engineering, Xi’an Shiyou University, Xi’an 710065, People's Republic of China; 2 College of Chemistry and Materials Science, Northwest University, Xi’an 710069, People's Republic of China; 3 No.203 Research lnstitute of Nuclear industry, Xianyang 712000, People's Republic of China

**Keywords:** QSPR, Polybrominated diphenyl ethers, Octanol/air partition coefficient, Molecular distance-edge vector index, Artificial neural network

## Abstract

**Background:**

The quantitative structure property relationship (QSPR) for octanol/air partition coefficient (*K*_OA_) of polybrominated diphenyl ethers (PBDEs) was investigated. Molecular distance-edge vector (MDEV) index was used as the structural descriptor of PBDEs. The quantitative relationship between the MDEV index and the lg*K*_OA_ of PBDEs was modeled by multivariate linear regression (MLR) and artificial neural network (ANN) respectively. Leave one out cross validation and external validation was carried out to assess the predictive ability of the developed models. The investigated 22 PBDEs were randomly split into two groups: Group I, which comprises 16 PBDEs, and Group II, which comprises 6 PBDEs.

**Results:**

The MLR model and the ANN model for predicting the *K*_OA_ of PBDEs were established. For the MLR model, the prediction root mean square relative error (*RMSRE*) of leave one out cross validation and external validation is 2.82 and 2.95, respectively. For the L-ANN model, the prediction *RMSRE* of leave one out cross validation and external validation is 2.55 and 2.69, respectively.

**Conclusion:**

The developed MLR and ANN model are practicable and easy-to-use for predicting the *K*_OA_ of PBDEs. The MDEV index of PBDEs is shown to be quantitatively related to the *K*_OA_ of PBDEs. MLR and ANN are both practicable for modeling the quantitative relationship between the MDEV index and the *K*_OA_ of PBDEs. The prediction accuracy of the ANN model is slightly higher than that of the MLR model. The obtained ANN model shoud be a more promising model for studying the octanol/air partition behavior of PBDEs.

## Background

Polybrominated diphenyl ethers (PBDEs) are a series of organobromine compounds that have been widely used as flame retardant in a variety of products, such as building materials, electronics, furnishings, coatings, plastics, etc [1,2]. Although the production of some PBDEs has been restricted under the Stockholm Convention since 2010, PBDEs have already become ubiquitous pollutants in the environment. They have been detected in many environmental compartments, such as air, water, soil, vegetations, animals and humans [3,4]. PBDEs have gained increasing attention because of their environmental persistence, bioaccumulation through the food chain, and potential risk to the human health [1,5,6]. PBDEs are lipophilic and semi-volatile compounds. The octanol/air partition of PBDEs may influence their fate, transport, and transformation in atmospheres [7-9]. The octanol/air partition coefficient (*K*_OA_), which is defined as the ratio of solute concentration in air versus octanol when the octanol/air system is at equilibrium, is a key parameter for describing the octanol/air partition of PBDEs between the atmosphere and organic phases such as soil, aerosol, vegetation and animals. Thus, a quantitative study on the *K*_OA_ of PBDEs is of great importance to understand the environmental fate of PBDEs. Many efforts have been made to determine the *K*_OA_ of PBDEs [7,9-11]. However, determining the *K*_OA_ of PBDEs is always a hard work due to the complexity of analytical methods, lack of chemical standards and high cost of experiments [4,12-15]. Thus, the quantitative structure-property relationship (QSPR) method, which is fast, easy-to-use and cost-effective [12,16,17], is always used to preliminary estimate the value of *K*_OA_ of PBDEs. Several QSPR models for the *K*_OA_ of PBDEs have been reported [12-15]. In these works, quantum chemical descriptors are used as the structural descriptor of PBDEs. However, developing a QSPR model based on quantum chemical descriptors is still a complex work, because the calculation and selection of structural descriptors are always time-consuming and complicated. It is still worthwhile to develop an easy-to-use QSPR model for the *K*_OA_ of PBDEs. Topological index is a kind of structural descriptor which has been widely used in the QSPR researches. It can effectively describe the structure of molecules without the detailed molecular orbital calculation and energy optimization. Topological index is useful because, despite its mathematical simplicity, it is able to differentiate molecules with different structures [18]. Therefore, the aim of our work is to investigate the QSPR model for the *K*_OA_ of PBDEs based on topological index. Molecular distance-edge vector (MDEV) index [19-21] was used as the structural descriptor of PBDEs. Multivariate linear regression (MLR) and artificial neural network (ANN) were employed to build the calibration model between the MDEV index and the *K*_OA_ of PBDEs.

## Results and discussion

Firstly, the MDEV index of the investigated 22 PBDEs was calculated. The obtained MDEV index is presented in Table 1. As shown in the table, the value of MDEV index for different PBDE molecules is different. It is demonstrated that MDEV index can describe the structural differences among these molecules. Thus, it is reasonable to use MDEV index as structural descriptor to develop the QSPR model of PBDEs.

**Table 1:** MDEV index of the investigated PBDEs

**No.**	**PBDE congeners**	***μ***_**1**_	***μ***_**2**_
1	2 -monobro	0	1.1111
2*	3 -monobro	0	1.0625
3	2,4 -dibro	0.0625	2.1511
4	2,4′ -dibro	0.0204	2.1511
5	2,6 -dibro	0.0625	2.2222
6*	3,4 -dibro	0.1111	2.1025
7	3,4′ -dibro	0.0156	2.1025
8	4,4′ -dibro	0.0123	2.0800
9	2,3,4 -tribro	0.2847	3.2136
10*	2,4,6 -tribro	0.1875	3.2622
11	2,4′,6 -tribro	0.1033	3.2622
12	3,3′,4 -tribro	0.1471	3.1650
13	3,4,4′ -tribro	0.1391	3.1425
14*	2,2′,4,4′ -tetrabro	0.2182	4.3022
15	2,3′,4,4′ -tetrabro	0.2498	4.2536
16	2,3′,4,6 -tetrabro	0.2587	4.3247
17	2,4,4′,6 -tetrabro	0.2407	4.3022
18*	3,3′,4,4′ -tetrabro	0.2862	4.2050
19	2,2′,3,3′,4 -pentabro	0.5478	5.3872
20	2,2′,4,4′,5 -pentabro	0.4127	5.3647
21	2,3′,4,4′,6 -pentabro	0.4230	5.3647
22*	2,2′,4,4′,5,5′ -hexabro	0.6276	6.4272

*The PBDE congeners in the test set (see text).

Secondly, two QSPR models were developed and investigated. One is MLR model and the other is L-ANN model. In order to assess the predictive ability of the developed models, two validation methods, leave one out cross validation and external validation, were conducted. The 22 PBDEs were randomly divided to two groups: Group I, which comprises 16 PBDEs, and Group II, which comprises 6 PBDEs (marked by asterisk in Tables 1 and 2).

**Table 2:** **Experimental and predicted lg*K***_**OA **_**of the investigated PBDEs**

**No.**	**Experimental lg*K***_**OA**_	**Predicted lg*K***_**OA**_		**Relative error (%)**	
		**MLR**	**ANN**	**MLR**	**ANN**
1	7.24	7.56	7.45	4.42	3.59
2*	7.36	7.38	7.40	0.27	0.54
3	8.37	8.43	8.43	0.72	0.36
4	8.47	8.46	8.45	−0.12	−0.12
5	8.12	8.54	8.50	5.17	5.05
6*	8.55	8.40	8.35	−1.75	−2.34
7	8.57	8.39	8.41	−2.10	−1.63
8	8.64	8.35	8.39	−3.36	−3.01
9	9.49	9.22	9.33	−2.85	−2.42
10*	9.02	9.53	9.44	5.65	4.66
11	9.28	9.54	9.49	2.80	2.26
12	9.61	9.34	9.37	−2.81	−2.81
13	9.68	9.32	9.35	−3.72	−3.82
14*	10.34	10.41	10.44	0.68	0.97
15	10.49	10.34	10.37	−1.43	−1.05
16	10.23	10.45	10.43	2.15	1.96
17	10.13	10.47	10.42	3.36	2.96
18*	10.7	10.27	10.30	−4.02	−3.74
19	11.14	11.38	11.29	2.15	2.15
20	11.28	11.35	11.36	0.62	0.27
21	11.52	11.28	11.35	−2.08	−1.39
22*	12.15	12.23	12.26	0.66	0.91

*The PBDE congeners in the test set (see text).

### MLR model

Generally, a simple model should always be chosen in preference to a complex model, if the latter does not fit the data better. Thus, we firstly investigate whether MLR can model the quantitative relationship between the MDEV index and the lg*K*_OA_ of these PBDEs. The MDEV index was used as independent variable and the lg*K*_OA_ was used as dependent variable to develop the model.

**Figure 1:**
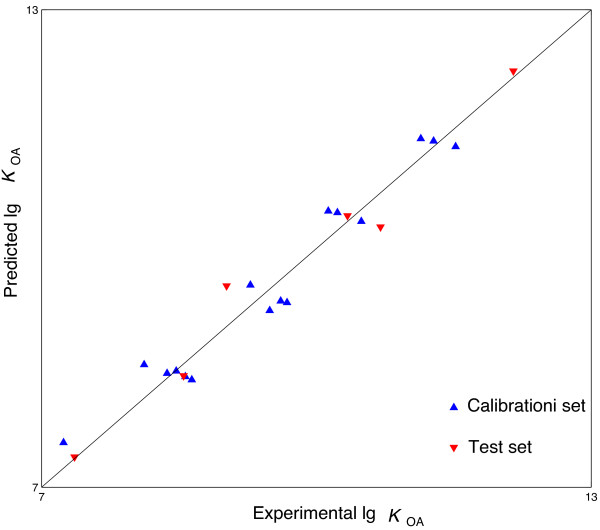
**Experimental lg** ***K***_**OA **_**versus the MLR model predicted lg** ***K***_**OA**_**.**

Firstly, leave one out cross validation was carried out. In the leave one out cross validation, the lg*K*_OA_ of all the samples in Group I was predicted in turn. The prediction procedure was performed 16 times. In each time, one sample was selected and used as the test set. The remaining 15 samples were used as training set to develop the regression model. The lg*K*_OA_ of the selected sample (test set) was then predicted with the obtained regression model. The result of leave one out cross validation is listed in Table 2. As shown in Table 2, the predicted lg*K*_OA_ are in good agreement with the experimental lg*K*_OA_. For the 16 samples of Group I, the prediction *RMSRE* is 2.82. In addition, the predicted lg*K*_OA_ were plotted versus the experimental lg*K*_OA_. The obtained plot is shown in Figure 1. The plot shows a linear relationship (*lgK*_*OA*,*pred*_ = 0.9635 *lgK*_*OA*,*exp*_ + 0.3573 with *R* = 0.9769) between the predicted and experimental lg*K*_OA_.

Subsequently, external validation was carried out to further assess the predictive ability of the MLR model. The regression model was developed by using all the 16 compounds in Group I. The obtained regression equation is:

(1) $$\text{lg}K_{\text{OA}}=0.7598\times \mu_{1}+0.9883\times \mu_{2}-6.3470$$

The *R*, Standard error of the estimate and *F* value of the regression model is 0.9844, 0.2340 and 202.46, respectively. Then, the lg*K*_OA_ of the six PBDEs in Group II was predicted by Equation 1. The prediction result is shown in Table 2 also. As shown in the table, the predicted lg*K*_OA_ are still in good agreement with the experimental lg*K*_OA_. The prediction *RMSRE* of the 6 PBDEs in Group II (marked by asterisk in Table 2) is 2.95. The plot of the predicted lg*K*_OA_ versus experimental lg*K*_OA_ is presented in Figure 1. As shown in Figure 1, there is a linear relationship (lg*K*_OA,pred_ = 0.9721 lg*K*_OA,exp_ + 0.2867 with *R* = 0.9836) between the predicted and experimental lg*K*_OA_.

The results of leave one out cross validation and external validation demonstrates that the MDEV index is quantitatively related to the *K*_OA_ of PBDEs. The established MLR model can describe the quantitative relationship between the MDEV index and *K*_OA_ of PBDEs. Compared with the QSPR models reported in the references [12-15], the obtained MLR model shows comparative prediction accuracy. MDEV index can be generated easier than quantum chemical descriptors. Thus, the developed MLR model is a reliable and easy-to-use QSPR model for predicting the *K*_OA_ of PBDEs.

### L-ANN model

L-ANN is an efficient and commonly used multivariate calibration method. Thus, we investigated whether a better model can be developed by using L-ANN appraoch. A 2-1 RBF-ANN (i.e. there are 2 nodes in the input layer and 1 node in the output layer) was used to model the quantitative relationship between the MDEV index and the lg*K*_OA_. The MDEV index was used as the input variable and the lg*K*_OA_ was used as the output variable.

**Figure 2:**
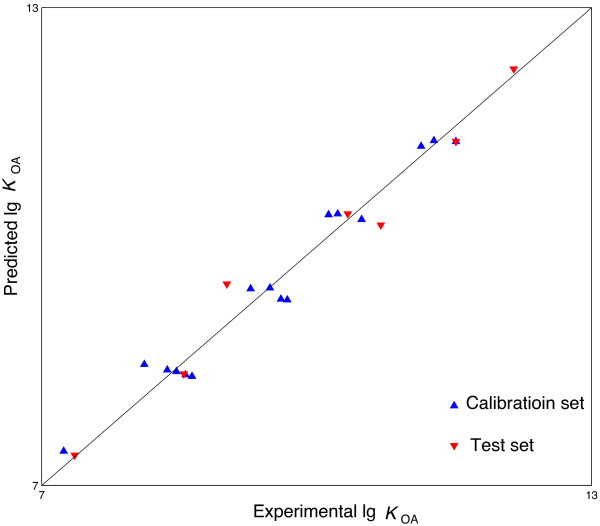
**Experimental lg** ***K***_**OA **_**versus the L-ANN model predicted lg** ***K***_**OA**_**.**

Group I was still used to carry out leave one out cross validation. In the leave one out cross validation, the lg*K*_OA_ of all the samples in Group I was predicted in turn. The prediction procedure was performed 16 times. In each time, one sample was selected and used as the test set. The remaining 15 samples were used as the calibration set to develop the network. Hence the 15 samples were randomly divided into a training set which includes 12 samples and a verification set which includes 3 samples. The lg*K*_OA_ of the selected sample (test set) was then predicted with the obtained network. The result of leave one out cross validation is listed in Table 2. For the 16 samples of Group I, the prediction *RMSRE* is 2.55. The plot of the predicted lg*K*_OA_ versus the experimental lg*K*_OA_ is presented in Figure 2. The regression equation and correlation coefficient between the predicted and experimental lg*K*_OA_ is lg*K*_OA,pred_ = 0.9731 lg*K*_OA,exp_ + 0.2640 and 0.9812 respectively.

Subsequently, the external validation was carried out by using all the 22 PBDEs. An L-ANN model was developed from the 16 PBDEs in Group II. In the training procedure, the verification set comprises three randomly selected samples and the rest 13 samples were used as the training set. The lg*K*_OA_ of the six PBDEs in Group I was then predicted with the obtained L-ANN model. The prediction result is presented in Table 2 also. The prediction *RMSRE* of the 6 PBDEs in Group II (marked by asterisk in Table 2) is 2.68. The plot of the predicted lg*K*_OA_ versus the experimental lg*K*_OA_ is shown in Figure 2. There is a linear relationship (lg*K*_OA, pred_ = 0.9854 lg*K*_OA, exp_ + 0.1535 with *R* =0.9864) between the predicted and experimental lg*K*_OA_. Obviously, the predicted lg*K*_OA_ is in good agreement with the experimental lg*K*_OA_. It is demonstrated that the quantitative relationship between the MDEV index and lg*K*_OA_ of PBDEs has been modeled well by L-ANN. Compared with the QSPR models reported in the references [12-15], the obtained L-ANN model shows comparative accuracy in predicting the lg*K*_OA_ of PBDEs. Obviously, it is a reliable and easy-to-use QSPR model for predicting the lg*K*_OA_ of PBDEs. In addition, the prediction result of the L-ANN model is slightly better than the result of the MLR model. Therefore, the established L-ANN model should be a more promising model for studying the octanol/air partition behavior of PBDEs.

## Experimental

### Data set

The MDEV index was calculated according to the approach presented in section “Methods: MDEV index”. The calculated MDEV index is listed in Table 1. The experimental lg*K*_OA_ of the 22 PBDEs listed in Table 2 is taken from references [12].

Root mean square relative error (*RMSRE*) was calculated to indicate the prediction performance of the obtained models. *RMSRE* is defined as:

(2) $$\text{RMSRE}=\sqrt{\frac{\sum {\left(RE_{i}\right)}^{2}}{n}}$$

where *RE*_*i*_ is the relative error of the *i*th sample, and *n* is the number of samples.

### Software

All the calculations were done with the subroutines developed under Matlab (Ver. 7.0). The computation was performed on a personal computer equipped with an i5-2450M processor. The used activation function of L-ANN is a linear function shown in Equation 5.

## Conclusion

Two QSPR models for the octanol/air partition of PBDEs were developed by using MLR and L-ANN respectively. The results of leave one out cross validation and external validation indicate that the obtained MLR model and L-ANN model are practicable for predicting the *K*_OA_ of PBDEs. It is demonstrated that the MDEV index is quantitatively related to the *K*_OA_ of PBDEs. MDEV index can be generated easier than quantum chemical descriptors. Thus, using MDEV index as structural descriptor is more convenient than using quantum chemical descriptor when developing the QSPR model for the *K*_OA_ of PBDEs. In addition, the result demonstrates MLR and L-ANN are both practicable for modeling the quantitative relationship between the MDEV index and *K*_OA_ of PBDEs. Compared with the established MLR model, the obtained L-ANN model shows slightly higher prediction accuracy. The obtained L-ANN model should be a more promising model for studying the octanol/air partition behavior of PBDEs.

## Methods

### MDEV index

In the calculation of MDEV index, a molecule is regarded as a geometric graph. Each non-hydrogen atom is regarded as a point and each chemical bond is regarded as an edge. The molecular structure of PBDEs can be encoded by the MDEV index of bromine atoms and benzene rings. If the relative electronegative of each bromine atom and benzene ring is defined as 1, the MDEV index of PBDEs can be defined as Equation 3:

(3) $$M_{\text{kl}}=\sum_{j\geq i}\frac{1}{d_{\text{ik},\text{jl}}^{2}}$$

(*k, l* =1,2 and *l* ≥ *k*)

where *k* and *l* denote the type of an atom (*k* =1 or *l* =1 denotes the bromine atom, and *k* =2 or *l* =2 denotes the benzene ring); *i* and *j* are the coding number of series number of a bromine atom or benzene ring in the molecular skeleton graph. In addition, *i* and *j* belong to the *k*th and *l*th type respectively. The *d*_*ik*_,_*jl*_ means the shortest relative distance between the *i*th and *j*th atom. For example, *d*_*i*1*,j*1_ denotes the nearest relative distance between the *i*th and *j*th bromine atom. The relative bond length between the two adjacent non-hydrogen atoms is defined as *d* = 1. According to Equation 3, there are three elements, *M*_11_, *M*_12_ and *M*_22_, in the MDEV index for a PBDE molecule. The three elements are usually noted as *μ*_1_, *μ*_2_ and *μ*_3_ respectively. For example, the MDEV index of 2,2’,4,4’-PBDE should be calculated as follows:

(4) $$\begin{array}{l} \mu_{1}=M_{11}={\left(\frac{1}{4}\right)}^{2}+{\left(\frac{1}{5}\right)}^{2}+{\left(\frac{1}{7}\right)}^{2}+{\left(\frac{1}{7}\right)}^{2}+{\left(\frac{1}{9}\right)}^{2}+{\left(\frac{1}{4}\right)}^{2}=0.2182\\ \mu_{2}=M_{12}={\left(\frac{1}{1}\right)}^{2}+{\left(\frac{1}{3}\right)}^{2}+{\left(\frac{1}{1}\right)}^{2}+{\left(\frac{1}{3}\right)}^{2}+{\left(\frac{1}{1}\right)}^{2}+{\left(\frac{1}{4}\right)}^{2}\\ \;+{\left(\frac{1}{1}\right)}^{2}+{\left(\frac{1}{4}\right)}^{2}=4.3022\\ \mu_{3}=M_{22}={\left(\frac{1}{1}\right)}^{2}=1 \end{array}$$

Obviously, the *M*_22_ of each PBDE is equal to 1. Thus, *μ*_1_ and *μ*_2_ were used to describe the structure of PBDEs.

### Artificial neural network

The theory of ANN has been elaborated in a lot of articles [21-28]. Hence, only a brief outline of ANN is presented here.

**Figure 3:**
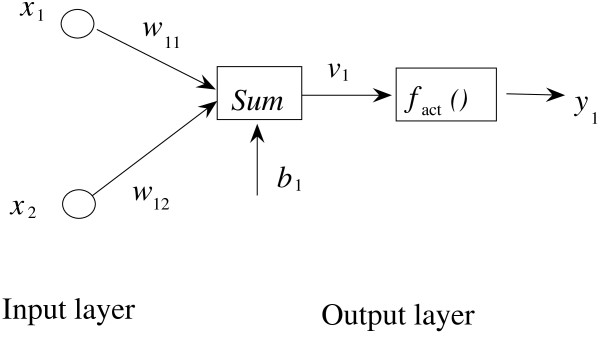
Architecture of linear artificial neural network.

ANN is a multivariate calibration method capable of modeling complex functions. The basic processing unit of ANN is the neuron (node). An artificial neural network comprises a number of neurons organized in different layers. Linear artificial neural network (L-ANN) [22-25] is a neural network having no hidden layers, but an output layer with fully linear neurons (that is, linear neurons with linear activation function). It is the simplest artificial neural network. In L-ANN, the neurons between the input and output layers fully connect, while the neurons in the same layer do not. Figure 3 illustrates the basic architecture of the used L-ANN.

In Figure 3, *x*_1_ and *x*_2_ are the input variables; *y*_1_ and *w*_1_ denotes the output variables and the element of connection weight matrix **W** respectively; *b*_1_ is the bias vector. The symbol *fact( )* means the activation function. Previous to training procedure, the input and output variables are normalized. When the network is executed, it multiplies the input variables by the weights matrix, and then adds the bias vector. The post synaptic potential (PSP) function of the neuron can be described as Equation 5:

(5) $$v_{j}=\sum_{i=1}^{n}x_{i}w_{\text{ij}}+b_{j}$$

Generally, the activation function used in L-ANN is a linear function which can be described as:

(6) $$y_{j}=v_{j}$$

Because there are no non-linear functions and hidden neurons in the network, L-ANN is ideal for dealing with linear problems. Actually, training a linear network means finding the optimal setting for the weight matrix **W** to minimize the root mean squared error (*RMSE*) of calibration set. In order to achieve this aim, the known samples which are used as calibraion set are generally divided into two parts: a training set and a verification set. The training set was used to calculate and adjust the network weights. The verification set was used to track the network's error performance, to identify the best network, and to stop training. The training should be stopped once deterioration in the verification error is observed. The optimal network parameters were selected according to the *RMSE* of verification set. The over-fitting and over-learning can be effectively avoided in this way. Although the verification set is used to identify the best network, actually, training algorithms do not use the verification set to adjust network weights. Standard pseudo-inverse linear optimization algorithm [22] is usually used to train the network. This algorithm uses the singular value decomposition technique to calculate the pseudo-inverse of the matrix needed to set the weights in a linear output layer, so as to find the least mean squared solution. Essentially, it guarantees to reach the optimal setting for the weights in the linear layer.

The main difference between MLR and L-ANN is the optimization algorithm. In MLR, the aim of least square algorithm is to minimize the sum of squared residuals of the training set. As for L-ANN, the aim of training algorithm is to minimize the *RMSE* of verification set [22].

### Leave one out cross validation

Leave one out cross validation [29] is a commonly used algorithm for estimating predictive performance of a multivariable calibration model. Usually, practical calibration experiments have to be based on a limited set of available samples. The idea behind the leave one out cross validation algorithm is to predict the property value of each sample in turn with the calibration model which is developed with the other samples. When applying the algorithm to a dataset with *N* samples, the calibration modeling is performed *N* times, each time using (*N*-1) samples for modeling and one sample for testing. Thus, the procedure of leave one out cross validation can be divided into *N* segment. In each segment *i* (*i* = 1, . . . , *N*), there are three steps: (1) taking sample *i* out as temporary ‘test set’, which is not used to develop the calibration model, (2) developing the calibration model with the remaining (*N*-1) samples, (3) testing the developed model with sample *i*, calculating and storing the prediction error of the sample.

#### External validation

External validation [26,30] is a algorithm which has been generally applied to estimating predictive performance of calibration models. When utilizing the algorithm, working dataset is split into two subsets: a calibration set, which is used to establish the calibration model, and a test set, which is employed to assess the predictive ability of the established calibration model. Herein, test set is designed to give an independent assessment of the predictive performance of the assed model. It is not used in establishing the calibration mdoel at all, and hence is independent of the calibration set. Generally, the samples in calibration set and test set are randomly selected from the working dataset.

## Competing interests

The authors declare that they have no competing interests.

## Authors’ contributions

LJ conceived of the study, carried out the calculation of structural descriptor and the model building, draft the manuscript. MMG and XFW participated in the design of the study and performed the statistical analysis, HL coordination and helped to draft the manuscript. All authors read and approved the final manuscript.
